# Physical seed damage, not rodent's saliva, accelerates seed germination of trees in a subtropical forest

**DOI:** 10.1002/ece3.11500

**Published:** 2024-07-17

**Authors:** Yunlong Zhu, Xifu Yang, Yuwei Teng, Zhenyu Wang, Zhibin Zhang

**Affiliations:** ^1^ State Key Laboratory of Integrated Management of Pest Insects and Rodents Institute of Zoology, Chinese Academy of Sciences Beijing China; ^2^ CAS Center for Excellence in Biotic Interactions University of Chinese Academy of Sciences Beijing China; ^3^ College of Life Science Jiangxi Normal University Nanchang China

**Keywords:** predation risk, rodents, rodents' saliva, seed fate, seed germination, seed traits, seedling reforestation

## Abstract

Many tree species adopt fast seed germination to escape the predation risk by rodents. Physical seed damage and the saliva of rodents on partially consumed seeds may act as cues for seeds to accelerate germination process. However, the impacts of these factors on seed germination rate and speed remain unclear. In this study, we investigated such impacts on the germination rate and speed (reversal of germination time) of four tree species (*Quercus variabilis*, *Q. serrata*, *Q. acutissima*, *Q. glauca*) after partial consumption by four rodent species (*Leopoldamys edwards*, *Niviventer fulvescens*, *N. confucianus*, *Apodemus draco*), through a series of experiments. We also examined how seed traits may affect the seed damage degree by rodents by analyzing the relationship between the germination rate and time of rodent‐damaged seed and the traits. We found that, artificially‐ and rodent‐damaged seeds exhibited a significantly higher seed germination rate and speed, compared to intact seeds. Also, the rodent saliva on seeds showed no significant effect on seed germination rate and speed. Furthermore, we observed significant positive correlations between several seed traits (including the seed mass, coat thickness, and protein content) and the seed germination rate and speed. These correlations are likely due to their beneficial traits countering seed damage by rodents. Overall, our results highlight the significant role of physical seed damage by rodents (rather than their saliva) in facilitating seed germination of tree species, and potential mutualism between rodents and trees. Additionally, our results may have some implications in forest restoration, such that intentionally sowing or dispersing slightly damaged seeds by humans or drones may increase the likelihood of successful seed regeneration.

## Graphical abstract

Our findings suggest that tree seeds can accelerate germination as a response to predation risk. Seeds with larger seed mass, thicker coats, higher protein content, and nondormant characteristics exhibit a positive effect on germination of damaged seeds. Overall, our results highlight the significant role of physical seed damage by rodents (rather than their saliva) in facilitating the seed germination of tree species.

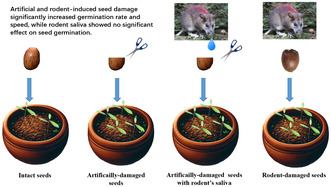

## 1 INTRODUCTION

Seed dispersal, germination, and seedling establishment are essential for forest regeneration (Chen et al., 2019; Nathan & Muller‐Landau, 2000; Yang, Gu, et al., 2022). Certain granivorous animal species such as rodents can significantly influence these process through their interactions with seeds (Chen et al., 2020; Nathan et al., 2008). Seeds are rich in nutrients, which can provide energy to rodents in winter and early spring when food is scarce (Smith & Reichman, 1984; Vander Wall, 1990). The rodents can either impose a predatory effect on tree by eating seeds, or a mutualistic effect by dispersing and caching seeds to a safe site for later seeding establishment (Mittelman et al., 2021; Zhang et al., 2024). Over‐predation of seeds by rodents would significantly reduce the seed regeneration of trees, and ultimately their fitness.

Under predation pressure by rodents, seeds have evolved into a series of strategies to escape rodent predation, such as resistance (Perea et al., 2011; Steele et al., 1993; Xiao et al., 2007), tolerance (Chen et al., 2012; Yang et al., 2023; Zhang et al., 2016; Zhang & Zhang, 2008), high capacity of regeneration (Cao et al., 2011) or seed cloning (Wang et al., 2021) or fast germination (Li et al., 2023). The tolerance mechanism allows seeds to maintain the ability to germinate and grow into seedlings even after being partially consumed, as long as the embryo remains intact (Mack, 1998; Vallejo‐Marin et al., 2006; Yi et al., 2021). Tolerance of seeds depends on the extent of damage, location of damage and species traits (Vallejo‐Marin et al., 2006). Vallejo‐Marin et al. (2006) discovered that seeds of *Aesculus californica* can still germinate successfully even after being bitten in half by animals. Furthermore, high capacity of regeneration allows seeds to germinate although the embryo is damaged or even removed (Cao et al., 2011). For instance, seeds of *Pittosporopsis kerrii* (including embryo‐removed seeds, all pruned seeds, and pruned roots) still retain a high regeneration capacity after rodent damage (Cao et al., 2011). In some cases, damaged seeds by rodents may even have a higher germination rate than intact ones because damaged fragments can produce more than one seedling (Cao et al., 2011; Yi et al., 2012, 2013). Seed cloning is also a unique strategy that any parts of the seed fragments can become an independent seedling after animal predation (Wang et al., 2021). This is, for example, the case that each fragment of a single seed *Garcinia xanthochymus* due to rodent or artificial damage can develop a single or even several seedlings (Wang et al., 2021). A recent study indicated that damaged seeds accelerated germination rate by perceiving change of plant hormones (Li et al., 2023). Such adaptive mechanisms indicate that seeds may have the capacity of countering seed damage by rodents, but it remains unclear how these damages could affect seed germination through distinct effect of physical damage and rodent's saliva left on the seeds.

Seed germination means nutrient loss for hoarding animals that rely on seeds as their main food reserves (Smith & Reichman, 1984; Vander Wall, 1990). Therefore, some rodent species (e.g., *Callosciurus erythraeus*, *Tamias striatus*, *Myoprocta exilis*) have evolved embryo‐removal or radicle‐pruning behaviors to reduce the possibility of seed germination or the delay of seed germination for their long‐term hoarding benefit (Cao et al., 2011; Elliott, 1978; Jansen et al., 2006; Xiao et al., 2009). Besides, rodents have evolved the behavior of preferring to eating germinated seeds rather than nongerminated ones and caching intact seeds for the purpose of a longer storage (Smallwood et al., 2001).

Seed damage by rodents may affect seed germination through their saliva. A few previous studies indicated that human saliva can inhibit seed germination. For example, Yardeni (1948) reported that human saliva inhibited the germination of wheat seeds and attributed this to the presence of antimicrobial substances in saliva. Kramer and Silberschmidt (1948) suggested that growth hormones in human saliva might be the factor responsible for inhibiting wheat seed germination. When rodents chew seeds, three paired salivary glands secrete saliva to soften and digest seeds, by changing the pH, bacterial composition, and hormone balance in the mouth (Dodds et al., 2015). But it is still not clear if rodents’ saliva could affect the seed germination of trees.

Seed traits (e.g., seed size, dormancy) may also affect the response of seed germination to seed damage. Previous studies have investigated the influence of seed traits, such as the size, coat thickness, tannin content, protein content, on seed germination (Baskin & Baskin, 2014; Cui et al., 2023; Xiao et al., 2022). Among these factors, seed size is the most influential (Passaretti et al., 2020). Larger seeds typically have more stored energy and water, which can lead to higher germination rates and faster emergence (Baskin & Baskin, 2014). Seeds with low moisture content and those with protective coats are more likely to germinate successfully due to their ability to avoid dehydration in hot and dry seeding sites (Correia et al., 2022; Dias Laumann et al., 2023). Thick seed coats can protect seeds from harsh environmental conditions but can also impede water uptake and gas exchange, potentially delaying or preventing germination (Bewley & Black, 2013). Seeds with higher protein content generally have better germination potential and produce more vigorous seedlings (Bera et al., 2023). In addition, other characteristics like dormancy mechanisms, seed shape, and the presence of micronutrients can also play a role in influencing the seed germination. The specific traits of seed may, therefore, influence the degree of damage by rodents on the seeds, and then affect their germination rates.

This study aims to investigate the distinct effects of physical damage and saliva of four species of rodents (*L. edwards*, *N. fulvescens*, *N. confucianus*, *A. draco*) on the seed germination rate of four oak species (*Quercus variabilis*, *Q. serrata*, *Q. acutissima*, *Q. glauca*) in the Dujiangyan region, Sichuan province, China. These four rodent species are responsible for the seed dispersal of these tree species (Yang et al., 2018). The impact of seed damage by rodents on the germination rate has two mechanisms. First, physical damage alone could cause a change in plant hormone which triggers seed germination (Li et al., 2023). Thus, artificially damaged seeds (i.e. cutting seeds into half) were used to test the distinct effect of physical damage by rodents. Second, rodents’ saliva may contact the seed kernel when rodent chew seeds, and then affect seed germination. Thus, artificially damaged seeds treated with rodents’ saliva were used to test the distinct effect of rodent's saliva on seed germination. The study focused on testing the following four specific hypotheses:

Hypothesis 1 Seeds may speed up germination to escape rodent predation when they are damaged by rodents. If this is true, we predict that physically damaged seeds would exhibit a higher germination rate and speed as compared to intact seeds.

Hypothesis 2 Physical damage is one aspect of how rodents' interations with seeds could affect germination rates, as it is a component of overall seed harm. If this is true, we predict that seeds (i.e. cutting seeds in half) would exhibit a higher germination rate and speed as compared to intact seeds.

Hypothesis 3 Rodents’ saliva may partially contribute to the effect of seed damage by rodents on seed germination since rodents’ saliva may contact the seed kernel, and then facilitate seed germination. If this is true, we predict that artificially damaged seeds treated with rodents’ saliva would have a higher germination rate and speed as compared to non‐saliva‐treated artificially damaged seeds.

Hypothesis 4 Seed traits that have a high tolerance or resistance to rodent predation may increase the germination of damaged seeds as they may help to reduce seed degree of damage. If this is true, we predict that seed size, seed coat thickness and high nutrient would have a positive correlation with the germination rate and speed of damaged seeds.

## 2 METHODS

### 2.1 Study site

The experiments were conducted at a field station located near the Prajna Temple (30°45′–31°45′ N, 103°47′–107°25′ E, elevation 700–1000 m) in the Puyang Town, Dujiangyan City, Sichuan Province, southwest China, from November 2019 to 2022. The climate of the study area is characterized as a humid mid‐subtropical climate, featuring short and mild winters, as well as long and relatively cool summers. The average temperature in the coldest month is 4.6°C (Chen, 2000). For details, see Yang et al. (2020) and Yang et al. (2018).

The local rodent community includes several species, such as South China field mouse (*Apodemus draco*), Chevrier's field mouse (*A. chevrieri*), Sichuan field mouse (*A. latronum*), Père David's vole (*Eothenomys melanogaster*), Chinese white‐bellied rat (*Niviventer confucianus*), Chestnut white‐bellied rat (*Niviventer fulvescens*), Edwards’ long‐tailed giant rat (*Leopoldamys edwards*), Brown rat (*Rattus norvegicus*), Himalayan field rat (*Rattus nitidus*), House mouse (*Mus musculus*), Eurasian harvest mouse (*Micromys minutus*), and others (Yang et al., 2018; Yang, Han, et al., 2022).

### 2.2 Study species

#### 2.2.1 Study oak species

In this study, we focused on the impacts of rodents on the germination of oak seeds from four oak species: Chinese cork oak (*Quercus variabilis*), Jolcham oak (*Quercus serrata*), Sawtooth oak (*Quercus acutissima*), and ring‐cupped oak (*Quercus glauca*). Mature seeds were collected from adult oak trees between September and November every year. The seeds were stored in plastic containers (60 cm × 30 cm × 10 cm) in the laboratory, with a 2 cm layer of sand on top serving as a drying agent. Different species of seeds were stored separately. To ensure the selection of sound oak seeds for the experiments, all the oak seeds were placed in water, and we removed any seeds that floated or were suspended (Gribko & Jones, 1995). Additionally, oak seeds showing signs of feeding damage, physical damage, or insect infestation were visually inspected and excluded from further analysis. The traits of these seed species are shown in Table S1 (See the Supplementary material section). Prior to this study, we conducted germination tests to categorize the seeds as dormant or nondormant, following the methods described in previous studies by Chen et al. (2020) and Baskin and Baskin (2014). Seeds were categorized as nondormant seeds if over 50% of the seeds successfully germinated within 4 weeks, while those with less than 50% germination were classified as dormant. Based on these standards, we classified *Q. variabilis*, *Q. serrata* as species with non‐dormant seeds, *Q. acutissima*, *Q. glauca* as species with dormant seeds.

#### 2.2.2 Study rodent species

Prior to the formal experiments, rodents were captured from the local forest using live traps (measuring L × W × H = 235 mm × 115 mm × 115 mm) made of steel wire mesh (diameter = 1 mm). We placed traps baited with Chinese chestnut (*Castanea mollissima*) seeds—a favorable food for rodents— in areas with evident rodent activity signs. Rodent activity leaves many traces, such as footprints, well‐trodden paths, fresh droppings, and gnawed seeds. We adjusted the trap density based on the intensity of these signs. In areas where rodent activity was clearly visible, we deployed 30 traps; In areas where rodent activity was not markedly noticeable, we deployed 10 traps. We set up 120 traps in all areas for 18 consecutive days. The traps were checked each morning for any captured rodents. Pregnant, immature, or unhealthy individual of all rodent species would be released back into their natural habitat. We determined pregnancy for female rodents based on the presence of vaginal suppository and the abdominal protuberance. Immature male or female rodents were identified by testicular descent or vaginal opening. Unhealthy individuals were identified based on symptoms of disease or distress, visible signs of injury or illness, and rodents' activity levels and reactions to external stimuli. Healthy adult rodents that we captured were identified, weighed, and assigned unique identification numbers for use in subsequent experiments. These adult rodents were housed individually in plastic boxes (*L* × *W* × *H* = 90 cm × 50 cm × 40 cm) for at least 2 weeks for rodents to acclimatize, following the methodology described by Wang et al. (2014). After being acclimatized, they were kept 30 days for the experiment. At the end of the Experiment 1 & 2, all rodents were released back into their original capture locations. The rodents in Experiment 3 were sacrificed under euthanized condition. All animal handling and care are in line with the guidance of the Animal Use and Care Committee, Institute of Zoology, Chinese Academy of Science. The certificate codes for staff conducting animal experiments presented by the Beijing Association for Experimental Animals after training is 1122042700104 for Yunlong Zhu, 1118091400032 for Xifu Yang and 1121111000108 for Yuwei Teng.

### 2.3 Germination experiments

To evaluate the effect of physical damage and saliva of rodents on the germination rate and speed of the oak seeds, we set‐up a germination experiment with intact and treated seeds. We planted the seeds in potted soil with each pot containing 5 seeds. The pots had a diameter of 12.5 cm and a depth of 11 cm. The seeds were planted at a burial depth of 2 cm, approximating the average burial depth of seeds hoarded by rodents (Xiao & Zhang, 2004). In Experiment 1, there were 264 pots and 1303 seeds in total. In Experiment 2, there were 60 pots and 300 seeds in total. In Experiment 3, there were 50 pots and 250 seeds in total. The germination progress of the planted seeds was regularly observed and recorded. Seed germination was defined as the emergence of any part of the seedling from the seed. Germinated seeds were carefully removed from the pots to minimize their influence on the nongerminated seeds. We also recorded the germination time of each seed to examine the relationship between germination time and seed traits (including seed mass, coat thickness, and protein content) of damaged seeds. In all experiments, injured oak seeds were referred to those damaged by rodents. We also used the data from Experiment 1 to test Hypothesis 4, which postulates that different seed traits demonstrating tolerance or resistance to rodent predation might affect the germination of damaged seeds. Seed traits of four species of oak seeds are obtained from a previous study (Yang et al., 2018). We used the following criteria to measure the germination metrics of oak seeds such as germination rate and time:

1. Germination rates (GRs), measured as the proportion of germinated oak seeds among all tested seeds.
2. Mean germination times (MGTs), measured as the average germination time of all germinated seeds during the experimentation period. The reversal of MGTs is referred to germination speed.

#### 2.3.1 Experiment 1. Effects of seed damage on seed germination

To test Hypothesis 1, 2 & 4 that artificial or rodent damage would speed up seed germination (higher germination rates but shorter germination times), we conducted experiments in Spring 2020, measuring the germination rate and time of partially‐damaged oak seeds of *Q. variabilis*, *Q. serrata*, *Q. acutissima*, and *Q. glauca*. These seeds were subjected to damage by three rodent species: *A. draco*, *A. chevrieri*, and *L. edwards*. Due to the dormancy of *Q. acutissima*, and *Q. glauca* oak seeds, we recorded their germination status weekly for 122 continuous days from January to May 2020. Prior to the experiments, the rodents were fasten for at least 6 h and then given the oak seeds in individual plastic boxes (*L* × *W* × *H* = 90 × 50 × 40 cm). Thus, both rodents and oak seeds were in the plastic boxes for the experiment. Twenty oak seeds were given for a *L. edwards* due to its large body mass (>200 g), 10 oak seeds were given for an *A. draco* and an *A. chevrieri*, considering their smaller body size and their daily intake, this experiment had 10 *A. draco*, 10 *A. chevrieri* and 5 *L. edwards* in controlled environments. The condition of the seeds was assessed based on feeding position and proportion to ensure that the seed embryos are not damaged. Seeds with half or more of the endosperm consumed were excluded from the germination tests based on feeding position and proportion. This daily intake information for individual *L. edwards*, as well as *A. draco* and *A. chevrieri*, was measured based on the number of seeds remaining after being eaten by rodents in our preliminary experiments. We used fresh intact seeds as the negative control group (NT group) serving as a reference to assess the natural conditions of seed germination without any interference from rodents or humans. This helps to establish a baseline of seed germination for making comparison between intact and treated seeds by rodents or artificial damage. We artificially damaged seeds, in which we manually cut the seed in half with normal saline to simulate the physical damage by rodent without effect of rodent's saliva, as positive control group (MJ group) to test the effect of physical seed damage and rodent's saliva on germination. Fresh oak seeds, which were handled by rodents (*L. edwards*, *A. draco*, *A. chevrieri*), and those that were still intact, were grouped into the LeT, AdT, and AcT categories to test the effects of other unknown factors except for seed damage. Fresh oak seeds handled by rodents (*L. edwards*, *A. draco*, and *A. chevrieri*), and those partially damaged were categorized into the LeJ, AdJ, and AcJ groups to test the effect of seed damage by rodents. The main distinction among these categories lies in whether the oak seeds were intact or damaged, and whether handled or not handled by rodents. Before planting into flower pots, all intact and partially damaged oak seeds were carefully cleaned with absorbent paper. The number of seeds involved in the Experiment 1 was shown in Supplement Table S9 and the eight treatment groups (NT, MJ, LeT, LeJ, AdT, AdJ, AcT, and AcJ) were shown below: NT, Naturally intact oak seeds; MJ, Mechanically damaged oak seeds by people with normal saline; LeT, Intact oak seeds handled by *L. edwards*; LeJ, Injured oak seeds by *L. edwards*; AdT, Intact oak seeds handled by *A. draco*; AdJ, Injured oak seeds by *A. draco*. AcT, Intact oak seeds handled by *A. chevri*; AcJ, Injured oak seeds by *A. chevrieri*.

#### 2.3.2 Experiment 2. Effects of mouth‐rinsed saliva on seed germination

The goal of this experiment was to explore how saliva of *L. edwards*, *N. fulvescens*, *N. confucianus*, and *A. draco* affect the germination of *Q. variabilis* and *Q. serrata* seeds. The experiment was conducted in autumn 2021, aiming to test Hypotheses (3) that rodent saliva may accelerate seed germination, aiming to seperate the effect of physical damage and saliva. Due to the nondormant characteristics of *Q. variabilis* and *Q. serrata*, seed germination occurred relatively quickly (Table S9), thus, from October to December 2021, initial monitoring of seed germination was conducted every 2–3 days, which was later adjusted to once a week, until 80% of the seeds had germinated and no further germination was observed in the subsequent 2 weeks. We used an 80% germination rate as the threshold to end monitoring because the remaining seeds might fail to germinate due to various reasons, such as dormancy mechanisms, germination potential. Due to insufficient numbers of *A. chevrieri*, we had to substitute *A. chevrieri* with *N. fulvescens* and *N. confucianus* in this experiment.

Before conducting the tests, all rodents were deprived of food for at least 6 hours and we preheated to a temperature of 37–38°C. We collected saliva by rinsing the mouths of each rodent with the pre‐heated saline water, and this process was repeated at least 10 times per rodent to obtain a sufficient amount of saliva extract. The saliva extract volume from each rodent species (five individuals in total of the experiment) exceeds 10 mL, and saliva from an individual rodent was spread over 10 seeds. We used cotton swabs to collect saliva and evenly spread it on the surface, ensuring that both sides of the seeds came into contact with rodent saliva. The number of seeds involved in the Experiment 2 were shown in Supplement Table S9 and the six treatment groups (NT, MJ, LeR, NfR, NcR, and AdR) were shown below: NT, Naturally intact oak seeds; MJ, Mechanically damaged oak seeds by people with normal saline; LeR, Injured oak seeds treated with mouth‐rinsed saliva of *L. edwards*; NfR, Injured oak seeds treated with mouth‐rinsed saliva of *N. fulvescens*; NcR, Injured oak seeds treated with mouth‐rinsed saliva of *N. confucianus*; AdR, Injured oak seeds treated with mouth‐rinsed saliva of *A. draco*.

#### 2.3.3 Experiment 3. Effects of gland‐rinsed saliva on seed germination

The experiment is similar to Experiment 2, excepting that we used saliva glands (*N. fulvescens*, *N. confucianus*, *A. draco*), aiming to test Hypotheses (3) in the autumn of 2022. We encountered a seed shortage and were unable to collect a sufficient quantity of seeds in local forest, forcing us to reduce the number of seeds all groups from 50 to 25 this year. Due to the excessively large size of *L. edwards* (body weight > 200 g), it was excluded in this test as it was difficult to be anesthetized. Saliva glands were surgically extracted from rodents for analysis. To minimize pains to rodents, we only used one individual for each rodent species group. The rodent was sufficiently anesthetized by injecting 1 g/24 ul ethyl bromide gradually. This experiment had one *N. fulvescens*, one *N. confucianus*, and one *A. draco*. Due to insufficient numbers of *A. chevrieri*, we had to substitute *A. chevrieri* with *N. fulvescens* and *N. confucianus* in this experiment. The salivary glands, including the parotid gland, submaxillary gland, and sublingual gland, were surgically removed. The collected salivary glands were then immersed in preheated saline solution at a temperature of 37°C. The bottle containing the salivary gland diluent was gently shaken, and after 5 min, the diluted saliva liquid was dropped into surface of cut oak seeds of *Q. variabilis* and *Q. serrata*. The oak seeds were cut into half to simulate physical seed damage by rodents and keep the half with embryo for experiment. Our objective was to maintain consistent levels of damage across all seeds to evaluate its influence on germination rates under controlled conditions. After the experiment, all animals raised were treated harmlessly. The number of seeds involved in Experiment 3 was shown in Supplement Table S9, the five treatment groups (NT, MJ, NfG, NcG, and AdG) were shown below: NT, Naturally intact oak seeds; MJ, Mechanically damaged oak seeds by people with normal saline; NfG, Injured oak seeds with gland‐rinsed saliva of *N. fulvescens*; NcG, Injured oak seeds with gland‐rinsed saliva of *N. confucianus*; AdG, Injured oak seeds treated with mouth‐rinsed saliva of *A. draco*.

### 2.4 Statistical analysis

R 4.3.2 and SPSS 22.0 for Windows were used for the data analysis. The Kaplan–Meier estimates (*p*.adjust = “BH”) of survivor functions for the germination time and germination rate were calculated using the survfit() function from the “survival” package in R. The survdiff() functions from “survminer” package were employed to assess the difference in probability of germinating seeds among treatment groups. The “rcorr” function (method = “pearson”) from the “Hmisc” package in R was employed to compute Spearman correlation coefficients and their corresponding significance levels (*p*‐values), illustrating the relationship between GRs (germination rates), MGTs (mean germination times), and seed traits. Effect of seed dormancy status on germination rate and time was analyzed using the Wilcoxon signed‐rank test in R.

## 3 RESULTS

### 3.1 Experiment 1. Effects of seed damage on seed germination rate

**FIGURE 1:**
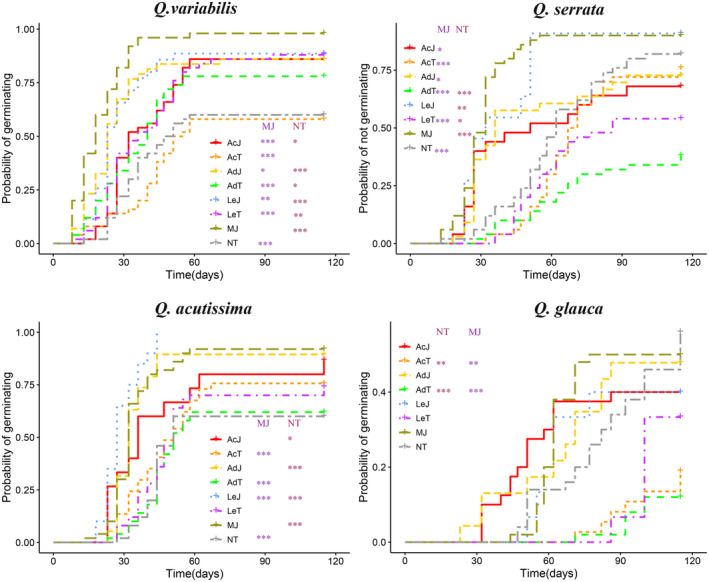
Effect of seed damage on probability of germination using Kaplan–Meier estimates of survivor functions for seeds of four tree species under different treatment groups including Naturally intact oak seeds (NT), Mechanically‐damaged oak seeds by people with normal saline (MJ), Intact oak seeds handled by *L. edwards* (LeT), Injured oak seeds by *L. edwards* (LeJ), Intact oak seeds handled by *A. draco* (AdT), Injured oak seeds by *A. draco* (AdJ), Intact oak seeds handled by *A. chevrieri* (AcT), Injured oak seeds by *A. chevrieri* (AcJ). *p < .05; **p < .01; and ***p < .001.

For *Q. variabilis*, Kaplan–Meier survival analysis showed statistically significant difference in the probability of germinating among groups (*χ* ^2^ = 113, *df* = 7, *p* < .001) (Figure 1). The probability of germinating of MJ group is significantly higher than those of NT, LeT, AdT, AcJ, AcT group (all *p* < .001), LeJ group (*p* < .01), and AdJ group (*p* < .05). The probability of germinating of NT group is significantly lower than those of MJ, LeJ, AdJ group (all *p* < .001), LeT group (*p* < .01), and AcJ or AdT group (all *p* < .05).

For *Q. serrata*, Kaplan–Meier survival analysis showed significant difference in the probability of germinating among groups (*χ* ^2^ = 105, *df* = 7, *p* < .001) (Figure 1). The probability of germinating of the MJ group is significantly higher than those of the NT, LeT, AdT, AcT group (all *p* < .001), as well as the AdJ or AcJ group (all *p* < .05). The probability of germinating of NT group is significantly lower than those of the MJ, AdT group (all *p* < .001) and LeT groups (*p* < .05), and LeJ group (*p* < .01).

For *Q. acutissima*, Kaplan–Meier survival analysis showed statistically significant difference in the probability of germinating among groups (*χ* ^2^ = 108, *df* = 7, *p* < .001) (Figure 1). The probability of germinating of MJ group is significantly higher than those of the NT, LeJ, AdT, AcT groups (all *p* < .001), as well as LeT group (*p* < .05). The probability of germinating of NT group is significantly lower than those of the MJ, LeJ, AdJ groups (all *p* < .001), and the AcJ group (*p* < .05).

For *Q. glauca*, Kaplan–Meier survival analysis showed statistically significant difference in the probability of germinating among groups (*χ* ^2^ = 32.3, *df* = 7, *p* < .001) (Figure 1). The probability of germinating of the MJ group is significantly higher than those of the AdT group (*p* < .001), and the AcT group (*p* < .01). The probability of germinating of the NT group is significantly higher than those of the AdT group (*p* < .001), and the AcT group (*p* < .01). The mean germination times (MGTs, days) of all oak seeds of four species in Experiment 1 were shown in Supplement Table S2. The germination rates (GRs, %) of four species of oak seeds in Experiment 1 were shown in Supplement Table S3.

In summary, artificially damaged seeds (MJ) had a significant higher probability of germinating than intact seeds (NT). Most rodent‐damaged seeds had a significant higher probability of germinating that NT seeds. It is notable that intact oak seeds handled (not damaged) by rodents showed a significant higher probability than intact oak seeds (NT) for *Q. variabilis*, not significant for *Q. acutissima* (no effect), and signigicant lower probability than intact oak seeds (NT) for *Q. glauca* (opposite effect) and *Q. serrata* (opposite effect).

#### 3.1.1 Relations between GR, MGT and seed traits

Using data from Experiment 1, we found the final germination rate (GR) of seeds by the end of the experiment are significantly and negatively correlated with starch content (*r* = −0.51, *p* < .001) but positively correlated with fresh weight (*r* = 0.59, *p* < .001), coat thickness (*r* = 0.59, *p* < .001), protein content (*r* = 0.53, *p* < .001), caloric value per seed (*r* = 0.59, *p* < .001); The mean seed germination times (MGTs) of all germinated seeds during the experimental periods are negatively correlated with fresh weight (*r* = −0.76, *p* < .001), coat thickness (*r* = −0.76, *p* < .001), protein content (*r* = −0.64, *p* < .001), caloric value per seed (*r* = −0.76, *p* < .001) ; no significant association between GRs and MGTs with fat content, tannin content, caloric value, body mass of rodents were found (*p* > .05; Table 1). The Wilcoxon signed‐ranked test indicated that there was signigicant difference in seed germination rates (V = 7443, *p* < .001) and mean germination time (V = 12880, *p* < .001) between dormant and non‐dormant seeds; non‐dormant seeds showed higher GRs but lower MGTs. Seed traits of four oak species in Experiment 1 were shown in Supplement Table S1.

**TABLE 1:** Correlations coefficients (r) between GR, MGT and seed traits.

Seed traits	GR	MGT
Fresh weight (g)	0.59***	−0.76***
Coat thickness (mm)	0.59***	−0.76***
Protein (%)	0.53***	−0.64***
Caloric value per seed (KJ)	0.59***	−0.76***
Fat (%)	0.092*	‐0.06
Starch (%)	−0.51***	0.37**
Tannin (%)	−0.14	‐0.099
Caloric value per gram (KJ/g)	−0.14	‐0.099
Rodent body mass (g)	‐0.034	0.059

*Note*: The positive or negative values indicate positive or negative correlation of seed trait with GR or MGT. *p < .05; **p < .01; ***p < .001.

In summary, seeds with a larger fresh weight, thicker coat, higher nutrition value or nondormancy are more tolerant or resistant to seed damage by rodents which result in a higher GRs and smaller MGTs.

### 3.2 Experiment 2. Effects of mouth‐rinsed saliva on seed germination

**FIGURE 2:**
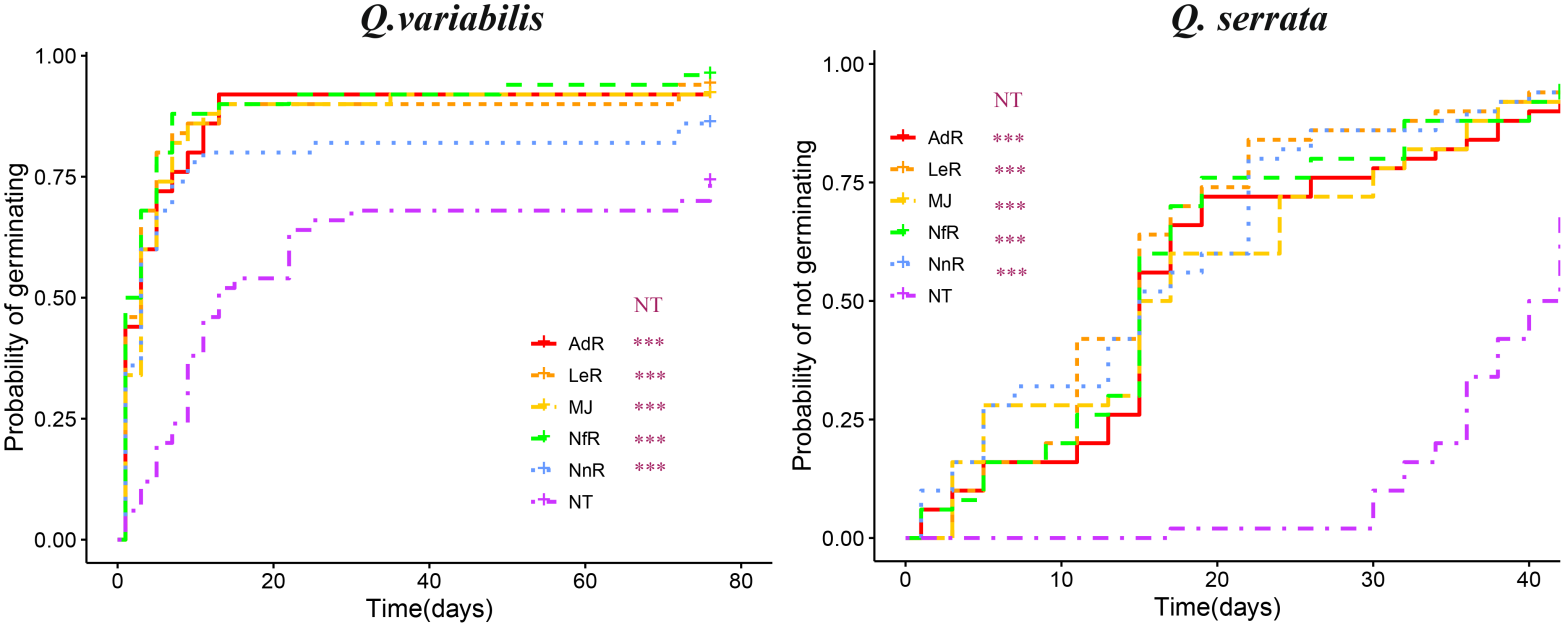
Effect of seed damage on probability of germination using Kaplan–Meier estimates of survivor functions for seeds of two tree species under different treatment groups including naturally Intact (NT), Mechanically‐damaged oak seeds with normal saline (MJ), Injured oak seeds with mouth‐rinsed saliva of *L. edwards* (LeR), Injured oak seeds with mouth‐rinsed saliva of *N. fulvescens* (NfR), Injured oak seeds with mouth‐rinsed saliva of *N. confucianus* (NcR), Injured oak seeds with mouth‐rinsed saliva of *A. draco* (AdR). ***p < .001.

For *Q. variabilis*, Kaplan–Meier survival analysis showed statistically significant difference in the probability of germinating among groups (*χ* ^2^ = 37.3, *df* = 5, *p* < .001) (Figure 2). The probability of germinating of NT group is significantly lower than those of MJ, NnR, NfR, AdR group (all *p* < .001). The probability of germinating between the MJ group and those of NnR, NfR, and AdR groups showed statistically nonsignificant differences (all *p* > .05).

For *Q. serrata*, Kaplan–Meier survival analysis showed statistically significant difference in the probability of germinating among groups (*χ* ^2^ = 59.7, *df* = 5, *p* < .001) (Figure 2). The probability of germinating of NT group is significantly lower than those of MJ, NnR, NfR, AdR groups (all *p* < .001). The probability of germinating between the MJ group and those of NnR, NfR, and AdR groups showed statistically nonsignificant differences (all *p* > .05). The mean germination times (MGTs, days) of oak seeds of two tree species in Experiment 2 were shown in Supplement Table S4. The germination rates (GRs, %) of seeds of two oak species in Experiment 2 were shown in Supplement Table S5.

In summary, artificially‐ or rodent‐damaged seeds showed a significant higher probability of germinating, but rodent mouse‐rinsed salivia did not significantly affect seed germination of artificially‐damaged seeds (MJ).

### 3.3 Experiment 3 effects of gland‐rinsed saliva on seed germination

**FIGURE 3:**
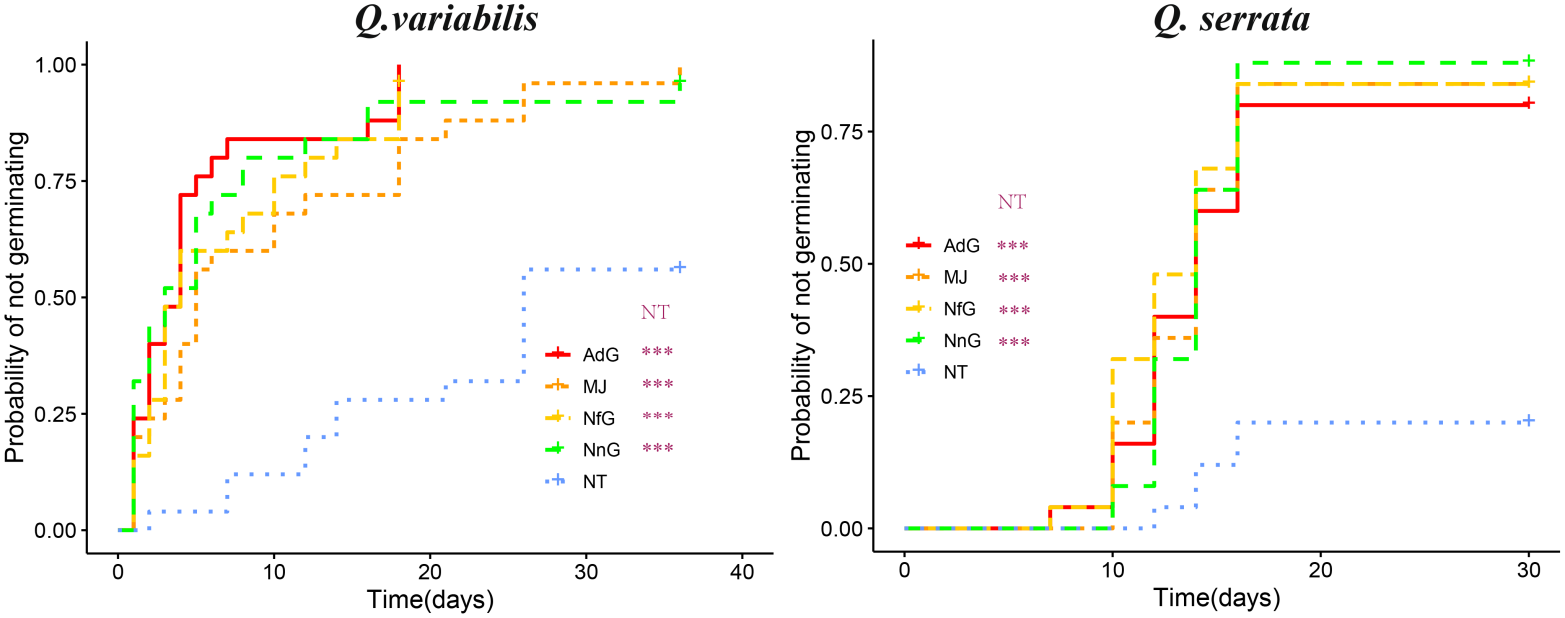
Effect of seed damage on probability of germination using Kaplan–Meier estimates of survivor functions for seeds of two tree species under different treatment groups Including Naturally intact oak seeds (NT), Mechanically‐damaged oak seeds with normal saline (MJ), Injured oak seeds with gland‐rinsed saliva of *N. fulvescens* (NfG), Injured oak seeds with gland‐rinsed saliva of *N. confucianus* (NcG), Injured oak seeds with gland‐rinsed saliva of *A.draco* (AdG). ***p < .001.

For *Q. variabilis*, Kaplan–Meier survival analysis showed statistically significant difference in the probability of germinating among groups (*χ* ^2^ = 45.2, *df* = 4, *p* < .001) (Figure 3). The probability of germinating of NT group is significantly lower than those of MJ, NnR, NfR, and AdR groups (all *p* < .001). The probability of germinating between the MJ group and those of MJ, NnR, NfR, and AdR groups showed statistically nonsignificant differences (all *p* > .05).

For *Q. serrata*, Kaplan–Meier survival analysis showed statistically significant difference in the probability of germinating among groups (*χ* ^2^ = 31.2, *df* = 4, *p* < .001) (Figure 3). The probability of germinating of NT group is significantly lower than those of MJ, NnR, NfR, and AdR groups (all *p* < .001). The probability of germinating between the MJ group and those of NnR, NfR, and AdR groups showed statistically nonsignificant differences (all *p* > .05). The mean germination times (MGTs, days) of seeds of two oak species in Experiment 3 were shown in Supplement Table S6. The germination rates (GRs, %) of seeds of two oak species in Experiment 2 were shown in Supplement Table S7.

In summary, artificially‐ or rodent‐damaged seeds showed a significant higher probability of germinating, but rodent gland‐rinsed saliva did not significantly affect seed germination of artificially damaged seeds (MJ).

## 4 DISCUSSION

Although fast seed germination is well known for seeds to escape animal predation, distinct impacts of physical seed damage and handling by rodents, as well as the effect of their saliva on the seed germination rates and times, have not been investigated. Our study demonstrates that seeds damaged (both artificially and by rodents) significantly accelerated germination, supporting our Hypothesis 1 & 2. In all saliva‐treatment tests, no significant effect of rodent saliva on the seed germination rate was found, not supporting our Hypothesis 3. The finding that a heavier fresh weight, thicker coat, higher nutrition value, or nondormancy are positively associated with higher germination rates and speeds supports our Hypothesis 4. Our results highlight the roles of physical damage and seed traits, as opposed to saliva stimulation, in enhancing seed germination following rodent damage, thereby supporting mutualism between rodents and tress. Our findings could have implications for seed reforestation, suggesting that sowing or spreading slightly damaged seeds in the field could be an effective strategy.

### 4.1 Effects of seed damage

The seed Tolerance Hypothesis (Vallejo‐Marin et al., 2006) suggests that seeds may develop a high level of tolerance to rodent damage as a strategy to avoid being fully preyed upon (Perea et al., 2011; Vallejo‐Marin et al., 2006; Xiao et al., 2007). Certain plants have evolved mechanisms to regenerate even after rodents remove their embryos. For example, Wang et al. (2021) demonstrated that the ability of *G. xanthochymus* seeds to clone themselves increased their capacity to withstand predation by animals. In this study, we observed that oak seeds exposed to damage by some rodent species and humans exhibited a higher germination rate, supporting our Hypothesis 1 and 2 that physical damage could accelerate seed germination. Our results are not only consistent with tolerance hypothesis that seed can germinate after partial seed consumption by rodents, but also indicate that rodent predation caused fast germination. Seed damage by rodents lead to increased seed germination rate or speed which may contribute to the mutualism between seeds and rodents, consistent with several previous studies (e.g., Cao et al., 2011; Fergnani et al., 2020; Li et al., 2023; Wang et al., 2021).

It is notable that intact oak seeds, handled but not damaged by rodents, showed a significant higher probability of germination than intact oak seeds (NT) for *Q. variabilis*. The reasons remain unknown. One plausible explanation is that the tested seeds may be of poor quality. Another possibility is that seeds might be immature. Further investigation is necessary to reconfirm the observation.

### 4.2 Effects of saliva of rodents

Kramer and Silberschmidt (1948) suggested that human saliva has an inhibitory effect on seed germination due to the presence of growth hormones and bacteriostatic substances. Additionally, seed germination also can be influenced by microorganisms, as highlighted by the research of Fu and Zou (2018). The mammalian oral cavity contains substances such as lysozyme and salivary proteins, lactoferrin, and peroxidase which can inhibit the growth of bacteria (Marcotte & Lavoie, 1998). Li et al. (2014) and Guo et al. (2020) have indicated that saliva‐treated plants may exhibit increased biomass, bud production, and activation of plant defenses to contribute to overall plant health and vigor. These prior studies indicate that animal saliva and related substances can trigger a series of physiological and biochemical reactions in plants. In our study, we did not find any significant facilitative or inhibitory effects of either mouth‐rinsed or gland‐rinsed saliva on seed germination of *Q. variabilis* and *Q. serrata*, not supporting our Hypothesis 3. It should be pointed out that the nonsignificant effects of saliva on seed germination may be caused by inappropriate conditions we used. Enzymes may work better in the mouth of rodents, while isolated saliva may be inactivated to some extent. Apart from temperature, the pH level may also be vital for enzyme activity (Grahame et al., 2015). Dilution of saliva in our study may alter pH level which result in inactivity of these enzymes. More work is needed to figure out the role of saliva in affecting the seed germination of plants. It is essential to understand the *ex vivo* condition of saliva of rodents before testing its effect on seed germination.

### 4.3 Effects of seed traits

Seed traits may influence the impact of seed damage on seed germination. Previous studies found that seeds with robust tolerance to animal predation often have a larger size and contain a nutrient‐rich endosperm or cotyledons, allowing partially damaged seeds to thrive in germination and eventually develop into seedlings (Mendoza & Dirzo, 2009; Perea et al., 2018). In our study, we observed that damaged seeds with heavier fresh weight or higher protein contents exhibited a higher germination rate or speed which is consistent with previous observations. We also discovered that damaged seeds with a higher seed coat thickness had a higher germnation rate, consisitent with previous observations in other system (e.g., Steele & Yi, 2020). Non‐dormant seeds often germinate quickly after falling down on the ground to escape rodent predation (e.g., Cao et al., 2011). In this study, we observed damaged seeds of nondormant seeds (e.g., *Q. variabilis* and *Q. serrata*) showed a higher germinate rate and speed as compared to dormant seeds (e.g., *Q. acutissima* and *Q. glauca*) which is also consistent with previous observations. These observations support our Hypothesis 4 that highly tolerant or resistant seed traits to rodent predation may increase the seed germination of damaged seeds.

### 4.4 Implication of the study

In summary, our study reveals that physical damage, rather than the saliva of rodents, accelerates the seed germination. Highly tolerant or resistant traits help seeds to counter for rodent predation by increasing the seed germination of damaged seeds.

Mutualism plays a crucial role in ecosystems, particularly in seed recruitment for many tree species, which heavily relies on balance between seed predation and seed dispersal by rodents (Zhang et al., 2021). Seed predation by rodents has been recognized to impose a negative effect on mutualism between rodents and trees. In our study, we discovered seed damage by rodents accelerated the seed germination of damaged seeds, suggesting rodent predation can promote mutualism between trees and rodents. This observation provides another empirical example of transformation from antagonism into mutualism (Zhang et al., 2021).

These findings carry implications for seedling production in forest restoration efforts. Intentionally sowing or dispersing slightly damaged seeds by humans or drones could increase the likelihood of successful seed regeneration. Future research endeavors should focus on unraveling the molecular and physiological mechanisms underlying rapid seed germination following rodent damage or handling.

## AUTHOR CONTRIBUTIONS

**Yunlong Zhu:** Conceptualization (equal); formal analysis (lead); methodology (lead); project administration (equal); visualization (lead); writing – original draft (lead); writing – review and editing (lead). **Xifu Yang:** Conceptualization (equal); funding acquisition (equal); methodology (equal); project administration (equal); writing – review and editing (equal). **Yuwei Teng:** Formal analysis (equal); methodology (equal); writing – review and editing (equal). **Zhenyu Wang:** Conceptualization (equal); funding acquisition (equal); methodology (equal); writing – review and editing (equal). **Zhibin Zhang:** Conceptualization (lead); project administration (lead); supervision (lead); writing – review and editing (lead).

## FUNDING INFORMATION

We would like to thank the support of the National Natural Science Foundation of China, Grant/Award Number: 32001123.

## CONFLICT OF INTEREST STATEMENT

The author declare that they have no conflict of interest.

## Supporting information


Table S1.
Table S2.
Table S3.
Table S4.
Table S5.
Table S6.
Table S7.
Table S8.
Table S9.

## Data Availability

Reviewer sharing link: https://datadryad.org/stash/share/‐PWiw8g0farQP4IvBjPI3iv5nqjT1lKSM1nYvA0r6ck
