# Supplementary material for: Physical seed damage, not rodent's saliva, accelerates seed germination of trees in a subtropical forest
Source: Ecol Evol. 2024 Jul 17;14(7):e11500. doi: 10.1002/ece3.11500 (PMC11254781; doi:10.1002/ece3.11500)
Supplement: Supplementary file 1 — Table S1. Table S2. Table S3. Table S4. Table S5. Table S6. Table S7. Table S8. Table S9. [file ECE3-14-e11500-s001.docx]

**Supplementary materials**

TABLE S1 Seed traits of four oak species in this study

| Seed traits | *Q. variabilis* | *Q. serrata* | *Q. acutissima* | *Q. glauca* |
| --- | --- | --- | --- | --- |
| Fresh mass(g) | 5.35 ± 0.84 | 1.46 ± 0.3 | 2.88 ± 0.59 | 0.90 ± 0.16 |
| Dry mass(g) | 3.85 ± 0.74 | 0.92 ± 0.20 | 1.84 ± 0.38 | 0.71 ± 0.12 |
| Coat thickness(mm) | 0.56 ± 0.09 | 0.39 ± 0.07 | 0.46 ± 0.11 | 0.31 ± 0.06 |
| Crude protein | 4.43 | 4.16 | 3.97 | 3.69 |
| Crude fat | 2.54 | 2.56 | 1.36 | 2.35 |
| Crude starch | 49.5 | 42.19 | 33.74 | 50.42 |
| Crude fiber | 2.08 | 2.18 | 1.80 | 2.02 |
| Tannin | 6.69 | 4.81 | 3.72 | 6.08 |
| Caloric value | 10.62 | 9.34 | 7.32 | 10.57 |
| Caloric value per seed | 32.08 | 6.17 | 10.03 | 5.92 |

Seed traits of four species of oak seeds are obtained from (Yang et al., 2018).

The analysis of nutritional composition in the seeds was conducted by the Grain Quality Supervision and Inspection Center of the Ministry of Agriculture, China, after collecting a mixture of fully developed seeds for each tree species.

Note: Comparison of scientific and common names

1. *variabilis -* Chinese cork oak*, Q. serrata -* Jolcham oak, *Q. acutissima -* Sawtooth oak, *Q. glauca -* ring-cupped oak.

Yang, X. F., Yan, C., Zhao, Q. J., Holyoak, M., Fortuna, M. A., Bascompte, J., Jansen, P. A., & Zhang, Z. B. (2018). Ecological succession succession drives the structural change of seed-rodent interaction networks in fragmented forests. Forest Ecology and Management, 419, 42-50. https://doi.org/10.1016/j.foreco.2018.03.023

TABLE S2 The mean germination time (MGTs, days) of seeds of four oak species in Experiment 1. For group abbreviations and full names, see: Table S8.

| Groups | *Q. Variabilis* | *Q. serrata* | *Q. acutissima* | *Q. glauca* |
| --- | --- | --- | --- | --- |
| NT | 35.20±0.83 | 58.04±2.51 | 42.50±0.31 | 81.27±3.59 |
| MJ | 19.36±0.83 | 29.51±0.79 | 33.25±1.20 | 61.87±0.78 |
| LeJ | 25.47±0.94 | 36.30±2.57 | 28.85±2.07 | 60.60±4.48 |
| LeT | 35.97±1.16 | 57.33±2.14 | 45.73±4.51 | 96.50±3.50 |
| AcJ | 35.27±1.50 | 39.69±3.52 | 42.93±8.03 | 49.02±2.80 |
| AcT | 38.79±1.72 | 65.10±3.05 | 43.41±1.75 | 92.07±3.67 |
| AdJ | 23.59±2.19 | 40.36±3.11 | 32.32±1.26 | 58.87±5.13 |
| AdT | 31.19±2.44 | 61.31±4.86 | 44.25±0.84 | 91.00±5.31 |

TABLE S3 The germination rates (GRs, %) of seeds of four oak species in Experiment 1. For group abbreviations and full names, see: Table S8.

| Groups | *Q. Variabilis* | *Q. serrata* | *Q. acutissima* | *Q. glauca* |
| --- | --- | --- | --- | --- |
| NT | 60.00±3.16 | 82.00±3.74 | 60.00±3.16 | 56.00±2.45 |
| MJ | 98.00±2.00 | 90.00±3.16 | 92.00±3.74 | 50.00±3.16 |
| LeJ | 88.57±5.35 | 100.00±0.00 | 100.00±0.00 | 40.00±6.67 |
| LeT | 88.00±2.00 | 56.00±4.00 | 68.005±3.74 | 10.00±3.16 |
| AcJ | 86.00±2.45 | 68.00±8.00 | 86.67±8.16 | 40.00±2.50 |
| AcT | 58.00±3.74 | 76.00±4.00 | 75.36±3.11 | 19.29±5.91 |
| AdJ | 86.11±2.11 | 38.00±3.74 | 90.00±6.12 | 47.00±6.63 |
| AdT | 78.00±3.74 | 72.86±8.30 | 62.00±4.99 | 12.00±2.00 |

TABLE S4 The mean germination time (MGTs, days) of seeds of two oak species in Experiment 2. For group abbreviations and full names, see: Table S8.

| Groups | *Q. Variabilis* | *Q. serrata* |
| --- | --- | --- |
| NT | 16.05±1.79 | 37.65±0.75 |
| MJ | 4.27±0.64 | 16.98±1.36 |
| LeR | 6.08±2.04 | 15.57±0.41 |
| NfR | 5.74±2.65 | 16.97±0.75 |
| NnR | 6.74±1.57 | 16.38±1.06 |
| AdR | 3.89±0.68 | 18.34±1.03 |

TABLE S5 The germination rates (GRs, %) of seeds of two oak species in Experiment 2. For group abbreviations and full names, see: Table S8.

| Groups | *Q. Variabilis* | *Q. Variabilis* |
| --- | --- | --- |
| NT | 74.00±5.10 | 80.00±3.16 |
| MJ | 92.00±2.00 | 92.00±2.00 |
| LeR | 94.00±2.45 | 96.00±2.45 |
| NfR | 96.00±2.45 | 96.00±2.45 |
| NnR | 86.00±2.45 | 98.00±2.00 |
| AdR | 92.00±3.74 | 96.00±2.45 |

TABLE S6 The mean germination times (MGTs, days) of seeds of two oak species in Experiment 3. For group abbreviations and full names, see: Table S8.

| Groups | *Q. Variabilis* | *Q. Variabilis* |
| --- | --- | --- |
| NT | 17.30±1.50 | 14.75±0.75 |
| MJ | 9.72±1.39 | 9.72±0.37 |
| NfG | 6.49±1.06 | 6.49±0.78 |
| NnG | 5.93±1.71 | 5.93±0.37 |
| AdG | 5.28±1.06 | 5.28±0.61 |

TABLE S7 The germination rates (GRs, %) of seeds of two oak species in Experiment 3. For group abbreviations and full names, see: Table S8.

| Groups | *Q. Variabilis* | *Q. Variabilis* |
| --- | --- | --- |
| NT | 56.00±9.80 | 20.00±6.32 |
| MJ | 100.00±0.00 | 84.00±4.00 |
| NfG | 96.00±4.00 | 84.00±9.80 |
| NnG | 96.00±4.00 | 80.00±10.95 |
| AdG | 100.00±0.00 | 88.00±8.00 |

Table S8 List of group abbreviations and full names

| Abbreviation | Full name |
| --- | --- |
| NT | Naturally intact oak seeds |
| MJ | Mechanically-damaged oak seeds artificially with normal saline |
| LeT | Intact oak seeds handled by *L. edwards* |
| LeJ | Injured oak seeds by *L. edwards* |
| AdT | Intact oak seeds handled by *A. draco* |
| AdJ | Injured oak seeds by *A. draco* |
| AcT | Intact oak seeds handled by *A. chevrieri* |
| AcJ | Injured oak seeds by *A. chevrieri* |
| LeR | Injured oak seeds treated with mouth-rinsed saliva of *L. edwards* |
| NfR | Injured oak seeds treated with mouth-rinsed saliva of *N. fulvescens* |
| NcR | Injured oak seeds treated with mouth-rinsed saliva of *N. confucianus* |
| AdR | Injured oak seeds treated with mouth-rinsed saliva of *A. draco* |
| NfG | Injured oak seeds with gland-rinsed saliva of *N. fulvescens* |
| NnG | Injured oak seeds treated with gland-rinsed saliva of *N. confucianus* |
| AdG | Injured oak seeds treated with gland-rinsed saliva of *A. draco* |

Table S9 Sample size of oak seeds and rodents in each group in Experiment 1, 2 and 3.

| Experiment 1. Effects of seed damage on seed germination | | | | | |
| --- | --- | --- | --- | --- | --- |
| Seed treatment | Sample size | | | | |
|  | Rodent | *Q.variabilis* | *Q. serrata* | *Q.acutissima* | *Q. glauca* |
| Naturally intact | / | 50 | 50 | 50 | 50 |
| Artificially damaged with saline | / | 50 | 50 | 50 | 50 |
| Injured seeds by *L. edwards* | 5 | 35 | 11 | 20 | 15 |
| Intact seeds handled by *L. edwards* | 5 | 50 | 50 | 50 | 50 |
| Injured seeds by *A. chevrieri* | 10 | 50 | 25 | 15 | 40 |
| Intact seeds handled by *A. chevrieri* | 10 | 50 | 50 | 37 | 37 |
| Injured seeds by *A. draco* | 10 | 43 | 33 | 19 | 23 |
| Intact seeds handled by *A. chevrieri* | 10 | 50 | 50 | 50 | 50 |
| Experiment 2. Effects of mouth-rinsed saliva on seed germination | | | | | |
| Naturally intact | / | 50 | 50 | / | / |
| Artificially damaged with saline | / | 50 | 50 | / | / |
| Injured seeds treated with mouth-rinsed saliva of *L. edwards* | 5 | 50 | 50 | / | / |
| Injured seeds treated with mouth-rinsed saliva of *N. fulvescens* | 5 | 50 | 50 | / | / |
| Injured seeds treated with mouth-rinsed saliva of *N. confucianus* | 5 | 50 | 50 | / | / |
| Injured seeds treated with mouth-rinsed saliva of *A. draco* | 5 | 50 | 50 | / | / |
| Experiment 3. Effects of gland-rinsed saliva on seed germination | | | | | |
| Naturally intact | / | 25 | 25 | / | / |
| Artificially damaged with saline | / | 25 | 25 | / | / |
| Injured oak seeds with gland-rinsed saliva of *N. fulvescens* | 1 | 25 | 25 | / | / |
| Injured oak seeds treated with mouth-rinsed saliva of *N. confucianus* | 1 | 25 | 25 | / | / |
| Injured oak treated with mouth-rinsed saliva of *A. draco* | 1 | 25 | 25 | / | / |

Note: The abbreviation and full name of the group name are shown below. For group abbreviations and full names, see: Table S8

**/**, No data.
